# EGFR Status by Immunohistochemistry in Triple-Negative Breast Cancer: An Evaluation of Prevalence and Association with Clinical and Pathological Parameters

**DOI:** 10.30699/ijp.2025.2042447.3363

**Published:** 2025-08-15

**Authors:** Elham Jafari, Mina Bagheri, Shahriar Dabiri, Aliasghar Tirgar, Vahid Moazed

**Affiliations:** 1 Pathology and Stem Cell Research Center, Department of Pathology, Afzalipour Faculty of Medicine, Kerman University of Medical Sciences, Kerman, Iran; 2 Department of Hematology and Oncology, Faculty of Medicine, Kerman University of Medical Sciences, Kerman, Iran

**Keywords:** Triple-negative breast cancer, EGFR, immunohistochemistry, tumor grade, prognostic marker

## Abstract

**Background & Objective:**

This study aimed to evaluate the expression patterns of epidermal growth factor receptor (EGFR) in patients with triple-negative breast cancer (TNBC) in Kerman, Iran, and investigate its association with various clinicopathological factors.

**Methods:**

A retrospective cross-sectional study was conducted on 54 TNBC patients diagnosed between 2013 and 2022. Immunohistochemistry (IHC) was performed to assess EGFR expression levels in tumor tissue samples. Patients were classified as EGFR-positive or EGFR-negative based on IHC staining results. Clinicopathological data, including age, tumor grade, vascular invasion, lymph node involvement, Ki-67 proliferation index, presence of necrosis, ductal carcinoma in situ (DCIS), and microcalcifications, were collected. Statistical analyses were performed to examine the association between EGFR expression and clinicopathological variables.

**Results:**

EGFR expression was positive in 61.1% of the patients. A significant association was found between EGFR positivity and higher tumor histologic grade (P = 0.045), with 69.7% of EGFR-positive patients exhibiting grade III tumors compared to 38.1% in the EGFR-negative group. Although not statistically significant, EGFR-positive patients tended to be younger (median age 44 years) than EGFR-negative patients (median age 52 years) (P = 0.157). The Ki-67 proliferation index was higher in EGFR-positive patients (median 60.0%) compared to EGFR-negative patients (median 47.5%).

**Conclusion:**

EGFR expression is significantly associated with higher tumor grade, suggesting a correlation with more aggressive tumor behavior. EGFR may serve as a potential prognostic marker and therapeutic target in TNBC. Further research with larger cohorts is recommended to validate these findings and explore the implications of EGFR-targeted therapies in TNBC management.

## Introduction

Breast cancer is a major public health concern in Iran, with studies indicating a rising incidence over recent decades (1). The mean age at diagnosis in Iranian women ranges from 47.4 to 51.2 years (2), which is notably lower than in developed countries such as the United Kingdom, where the highest incidence is observed after age 55 (3).

Triple-negative breast cancer (TNBC) is defined by the absence of estrogen receptor (ER), progesterone receptor (PR), and human epidermal growth factor receptor 2 (HER2) expression (4). TNBC accounts for approximately 20% of newly diagnosed breast cancer cases and is increasing in prevalence (5). Among Iranian patients diagnosed with breast carcinoma, the reported prevalence of TNBC is approximately 14% (6).

Gene expression profiling has revealed the molecular heterogeneity of TNBC and enabled the classification of this subtype into biologically and clinically relevant groups. In a landmark study, Lehmann et al. identified six TNBC molecular subtypes based on gene expression: basal-like 1 (BL1), basal-like 2 (BL2), immunomodulatory (IM), mesenchymal (M), mesenchymal stem-like (MSL), and luminal androgen receptor (LAR) (7). BL1 and BL2 are enriched for genes involved in cell cycle regulation and proliferation. The IM subtype is associated with immune signaling pathways, while the M and MSL subtypes are characterized by expression of genes related to epithelial-mesenchymal transition and growth factor signaling. The LAR subtype is defined by androgen receptor pathway activation (7). Histologically, TNBC frequently presents as high-grade invasive ductal carcinoma with basal-like features, including expression of basal cytokeratins and epidermal growth factor receptor (EGFR) (8).

Subsequent refinements to this classification have been proposed. Lehmann et al. later condensed the original six subtypes into four (BL1, BL2, M, LAR), after concluding that the IM and MSL groups primarily reflected tumor-infiltrating lymphocytes and tumor-associated stromal cells, rather than intrinsic tumor subtypes (9). Similarly, Burstein et al. described four subtypes—Basal-like Immune Activated, Basal-like Immune Suppressed, Mesenchymal, and Luminal AR—that largely overlap with Lehmann’s categories (10). These subtypes continue to be validated in ongoing studies, with the goal of integrating them into clinical practice to guide treatment selection (8).

EGFR, a receptor tyrosine kinase of the ErbB family, plays a critical role in angiogenesis, cell proliferation, and metastasis. It is frequently overexpressed in TNBC, particularly in basal-like subtypes, and is associated with poor prognosis and reduced survival (11,12). At the molecular level, EGFR signaling promotes tumor cell proliferation, invasion, and metastasis in TNBC, and may also contribute to drug resistance. This oncogenic activity is often potentiated by cross-talk with other receptors, such as insulin-like growth factor receptor 1 (IGF-1R) (13–15).

Preclinical studies have shown that targeting EGFR with tyrosine kinase inhibitors or monoclonal antibodies can suppress tumor growth and decrease cell viability in TNBC models (16). Despite this promising rationale, clinical trials of EGFR inhibitors in unselected TNBC populations have yielded limited success (17,18). This limited efficacy highlights the importance of identifying predictive biomarkers, such as EGFR mutations or gene amplification, to select patients who may benefit from EGFR-targeted therapies (19,20). Furthermore, the strong correlation between EGFR overexpression and risk of relapse underscores the relevance of EGFR as a potential therapeutic target in TNBC (21). High EGFR expression is also significantly associated with higher tumor grade and advanced stage at diagnosis, and is linked to worse disease-free and overall survival (12,22,23).

Given the clinical significance of EGFR in TNBC and the limited data from Iran, our study aimed to evaluate EGFR expression patterns among patients with TNBC in Kerman, Iran.

## Materials and Methods

### Participants

This retrospective cross-sectional study was conducted at Kerman University of Medical Sciences over a 10-year period from 2013 to 2023. The primary aim was to evaluate the prevalence of epidermal growth factor receptor (EGFR) expression using immunohistochemistry (IHC) among patients diagnosed with triple-negative breast cancer (TNBC) and assess its correlation with clinicopathological variables. Eligible participants included female patients diagnosed with breast cancer confirmed as triple-negative subtype by IHC analysis of core needle biopsy samples. Cases lacking sufficient data were excluded and replaced with new eligible participants.

### Histopathological Assessment

Tumor specimens were assessed for estrogen receptor (ER), progesterone receptor (PR), HER2 status, and Ki-67 proliferation index. Additional histopathological features evaluated included tumor type, Nottingham histologic grade, presence of lymphovascular invasion, lymphocyte host response, in situ components, multifocality, and lymph node involvement. The lymphocyte host response was graded as mild (A), moderate (B), or severe (C) according to previously established criteria (24).

### Immunohistochemistry Protocol

IHC staining was performed on formalin-fixed, paraffin-embedded (FFPE) tissue sections to detect EGFR antigen expression. Tissue sections of 2–5 μm thickness were obtained using a rotary microtome (model CUT 4050; microTec Laborgeräte GmbH, Walldorf, Germany) and mounted on positively charged glass slides. Standard protocols were used for deparaffinization and rehydration. Endogenous peroxidase activity was blocked with 3% hydrogen peroxide for 10 minutes.

Antigen retrieval was performed in a pressure cooker using EDTA buffer (pH 9.0). After washing in phosphate-buffered saline (PBS), sections were incubated with primary antibodies diluted 1:100. Primary antibodies included anti-EGFR (Shanghai Long Island Antibody Diagnostica Inc., catalog no. R-023B), anti-p53 (ScyTek, USA, A00109), anti-Bcl-2 (ScyTek, USA, A00119), and anti-Ki-67 (ScyTek, USA, A00095). Secondary antibody incubation and DAB chromogenic detection were performed following the manufacturers’ protocols, and all slides were counterstained with hematoxylin.

The specificity of IHC staining was verified using negative controls in which the primary antibody was omitted. All slides were scored independently by two blinded pathologists using an Olympus CX41 microscope.

### Interpretation of EGFR Staining

Only invasive tumor areas were evaluated for EGFR expression; normal tissue, ductal carcinoma in situ (DCIS), and necrotic regions were excluded. Microscopic evaluation was performed at ×400 magnification to ensure accurate visualization of EGFR staining.

Tumor cells were considered EGFR-positive if they exhibited clear membrane and/or cytoplasmic brown staining. The percentage of EGFR-positive tumor cells was calculated by dividing the number of stained cells by the total number of tumor cells within the field of analysis.

**Fig 1:**
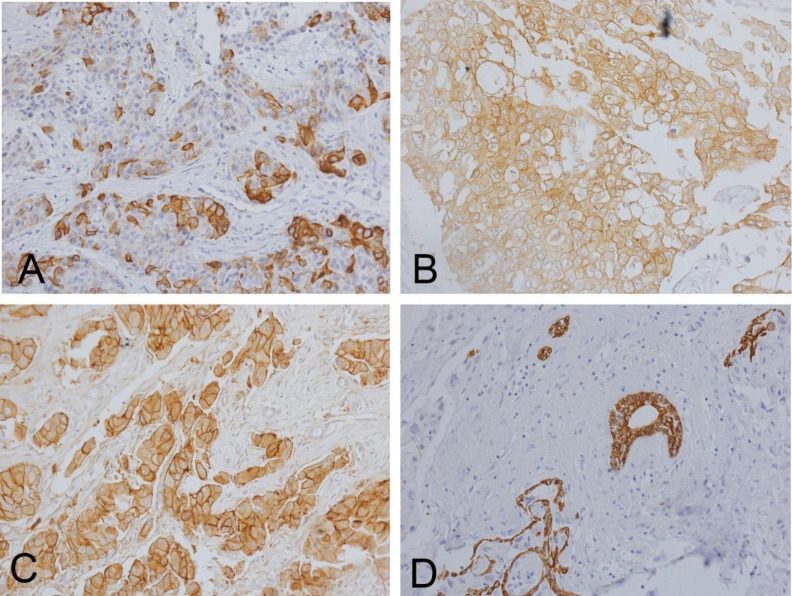
Examples of EGFR staining assessed through the H & E method, accompanied by microscopic examination and subsequent classification. Panel (A) represents low levels of EGFR staining, panel (B) demonstrates intermediate levels, and panel (C) exhibits high levels of EGFR staining. D represent negative control. All images were captured at ×400 magnification.

EGFR expression was categorized according to Zgura et al. (2018) (25) as follows (illustrated in Figure 1):

1. **Low EGFR expression**: 0–10% of tumor cells positive (Fig. 1A)
2. **Intermediate EGFR expression**: 10–40% of tumor cells positive (Fig. 1B)

**High EGFR expression**: 40–90% of tumor cells positive (Fig. 1C)

### Statistical Methods

Descriptive statistics were used to summarize the data. Continuous variables were presented as mean ± standard deviation (SD), and categorical variables were expressed as frequencies and percentages. The Shapiro–Wilk test was applied to assess the normality of continuous data distributions.

For normally distributed variables, comparisons between groups were made using the Student *t* test or one-way analysis of variance (ANOVA), as appropriate. The chi-square test was used to evaluate associations between categorical variables. For non-normally distributed data, the Mann–Whitney *U* test or Kruskal–Wallis test was applied.

All statistical analyses were performed using Stata Statistical Software, Release 17 (StataCorp LLC, College Station, TX, USA). A *P* value < .05 was considered statistically significant.

## Results

A total of 54 patients with triple-negative breast cancer (TNBC) were evaluated. The mean age was 48.5 ± 12.3 years, with 57.4% of patients aged 50 years or younger.

Immunohistochemical (IHC) analysis revealed that 61.1% of tumors were EGFR-positive. The distribution of EGFR staining intensity was as follows: 0 (negative), 38.9%; 1+, 38.9%; 2+, 9.3%; and 3+, 13.0%.

Histopathological grading showed that 57.4% of tumors were grade III and 42.6% were grade II. Vascular invasion was identified in 81.5% of cases. The lymphocyte host response was predominantly mild (type A) in 72.2% of patients, followed by moderate (type B) in 22.2% and severe (type C) in 5.6%.

Lymph node involvement was present in 25.9% of patients, while 66.7% had no lymph node metastasis. The mean Ki-67 proliferation index was elevated at 51.7% ± 17.3%. Tumor necrosis was observed in 57.4% of cases, ductal carcinoma in situ (DCIS) components in 25.9%, and microcalcifications in 3.7%.

When comparing EGFR-positive and EGFR-negative groups, the median age was 44 and 52 years, respectively (*P* = .157). A trend toward higher EGFR expression among younger patients was noted: 69.7% of EGFR-positive patients were ≤50 years, compared to 38.1% in the EGFR-negative group.

A statistically significant association was found between EGFR expression and tumor histologic grade (*P* = .045). Among EGFR-positive tumors, 69.7% were grade III, compared to 38.1% in EGFR-negative cases.

No significant differences were identified between EGFR expression and nuclear grade (*P* = .435), vascular invasion (*P* = .162), lymphocyte host response (*P* = .364), lymph node involvement (*P* = .557), Ki-67 index (*P* = .448), tumor necrosis (*P* = .754), presence of DCIS (*P* = .547), or microcalcifications (*P* = 1.000).

**Table 1:** Clinicopathological data of the studied patients.

Characteristics	Subgroup	Overall
		54
Age, mean (SD)		48.5 (12.3)
EGFR Score, n (%)	**0**	21 (38.9)
	**1**	21 (38.9)
	**2**	5 (9.3)
	**3**	7 (13.0)
Age group (year), n (%)	**≤ 50 years**	31 (57.4)
	**> 50 years**	19 (35.2)
	**Unknown**	4 (7.4)
EGFR Class, n (%)	**Negative**	21 (38.9)
	**Positive**	33 (61.1)
Tumor grade, n (%)	**II**	23 (42.6)
	**III**	31 (57.4)
Vascular invasion, n (%)	**No**	10 (18.5)
	**Yes**	44 (81.5)
Lymph host response, n (%)	**A**	39 (72.2)
	**B**	12 (22.2)
	**C**	3 (5.6)
Lymph node invasion, n (%)	**No**	36 (66.7)
	**Yes**	14 (25.9)
	**Unknown**	4 (7.4)
Ki-67, mean (SD)		51.7 (17.3)
Necrosis, n (%)	**No**	23 (42.6)
	**Yes**	31 (57.4)
DCIS, n (%)	**No**	40 (74.1)
	**Yes**	14 (25.9)
Microcalcification, n (%)	**No**	52 (96.3)
	**Yes**	2 (3.7)

**Table 2:** Comparison of clinicopathological features based on EGFR staining.

Grouped by EGFR Staining Status					
Characteristic	Subgroup	Overall	No	Yes	P-Value
		54	21	33	
Age, median [min,max]		46.5 [28,77]	52[28,77]	44 [28,71]	0.157
Agegroup, n (%)	≤ 50 years	31 (57.4)	8 (38.1)	23 (69.7)	0.051
	> 50 years	19 (35.2)	10 (47.6)	9 (27.3)	
	Unknown	4 (7.4)	3 (14.3)	1 (3.0)	
Tumorgrade, n (%)	II	23 (42.6)	13 (61.9)	10 (30.3)	0.045
	III	31 (57.4)	8 (38.1)	23 (69.7)	
Vascularinvasion, n (%)	No	10 (18.5)	6 (28.6)	4 (12.1)	0.162
	Yes	44 (81.5)	15 (71.4)	29 (87.9)	
Lymphhostresponse, n (%)	A	39 (72.2)	16 (76.2)	23 (69.7)	0.364
	B	12 (22.2)	3 (14.3)	9 (27.3)	
	C	3 (5.6)	2 (9.5)	1 (3.0)	
Lymph node invasion, n (%)	No	36 (66.7)	13 (61.9)	23 (69.7)	0.557
	Yes	14 (25.9)	7 (33.3)	7 (21.2)	
	Unknown	4 (7.4)	1 (4.8)	3 (9.1)	
Ki-67, median [min,max]		55.0 [25.0,80.0]	47.5 [35.0,70.0]	60.0 [25.0,80.0]	0.448
Necrosis, n (%)	No	23 (42.6)	10 (47.6)	13 (39.4)	0.754
	Yes	31 (57.4)	11 (52.4)	20 (60.6)	
DCIS, n (%)	No	40 (74.1)	17 (81.0)	23 (69.7)	0.547
	Yes	14 (25.9)	4 (19.0)	10 (30.3)	
Microcalcification, n (%)	No	52 (96.3)	20 (95.2)	32 (97.0)	1.000
	Yes	2 (3.7)	1 (4.8)	1 (3.0)	

## Discussion

This study evaluated EGFR expression patterns in patients diagnosed with TNBC in Kerman, Iran, and explored its associations with various clinicopathological parameters. Our findings revealed a high prevalence (61.1%) of EGFR positivity among TNBC cases, aligning with prior research. For example, Atik et al. reported EGFR positivity in 87.2% of TNBC cases compared to only 8% in non-TNBC tumors (26). Similarly, Changavi et al. found EGFR expression in 89.47% of triple-negative cases (27), supporting the consistent association between EGFR overexpression and TNBC across diverse populations.

The significant association between EGFR expression and higher tumor grade observed in our cohort underscores its potential role as a prognostic biomarker. This is consistent with Lauvanya et al. (2024), who reported that EGFR expression—detected in 48.9% of TNBC cases—was plausibly associated with aggressive clinicopathological features such as higher histological grade and larger tumor size (28). Likewise, Samarnthai et al. (2024) identified the absence of EGFR expression as an independent predictor of reduced disease-free survival (DFS) and overall survival (OS) in TNBC patients (29), further reinforcing EGFR’s prognostic relevance.

Although some studies have reported lower rates of EGFR expression, such as Salman et al. (2022), who found a 28% positivity rate in Iraqi TNBC cases (31), most literature confirms a significant correlation between EGFR overexpression and poor clinical outcomes, including increased risk of metastasis and mortality. Discrepancies in EGFR prevalence may be attributed to differences in population genetics, immunohistochemistry (IHC) techniques, antibodies used, or thresholds for EGFR positivity.

Our data also revealed that TNBC predominantly affected younger patients and was frequently associated with grade III tumors. These findings mirror those of Kanapathy Pillai et al. (32), who reported a similar age distribution and histological grade among TNBC patients in Malaysia. The high Ki-67 proliferation index in our cohort (mean: 51.7%) supports the aggressive nature of TNBC and aligns with existing reports emphasizing the diagnostic and prognostic utility of proliferation markers (33,34).

The correlation between EGFR positivity and histological grade observed in our cohort is consistent with the findings of Sood and Nigam (35), who also noted a significant association. Moreover, Wang et al. found that EGFR expression following neoadjuvant chemotherapy was associated with poorer DFS (36), highlighting EGFR's prognostic utility beyond diagnostic assessment.

Despite the strong rationale for EGFR-targeted therapies in TNBC, clinical trials have yielded modest results. Carey et al. reported limited efficacy of EGFR inhibitors in unselected TNBC populations (17), suggesting that molecular heterogeneity and resistance mechanisms likely contribute to variable therapeutic responses. Our findings, demonstrating a high prevalence of EGFR positivity, underscore the need for biomarker-driven patient selection in future clinical trials.

Given the established heterogeneity of TNBC subtypes, as characterized by Lehmann et al. and Burstein et al. (7,10), it is plausible that EGFR expression differs among molecular subtypes. These nuances support the need for personalized treatment approaches that incorporate EGFR status alongside other molecular markers to improve patient stratification and treatment efficacy.

The predominance of high-grade tumors and diagnosis at a younger age observed in our study is concerning, given their established associations with poor prognosis. These data highlight the urgent need for early detection programs and the development of novel targeted therapies, particularly in regions with a high burden of aggressive breast cancer phenotypes.

**Limitations** of our study include its retrospective design and potential selection biases. Furthermore, while our sample size was sufficient for initial analysis, it may not fully capture the molecular diversity of TNBC. Larger, prospective studies are warranted to validate these findings and further explore the clinical implications of EGFR expression.

## Conclusion

This study highlights the high prevalence of EGFR expression in TNBC among Iranian patients, consistent with global data. The significant association between EGFR positivity and higher tumor grade underscores its potential as both a prognostic biomarker and a therapeutic target. Future research should focus on patient stratification based on EGFR status and the clinical evaluation of EGFR-targeted therapies to improve outcomes in this aggressive breast cancer subtype.

## Data Availability

There is no additional data separate from available in cited references.
